# Detection of Cell Surface Ligands for Human Synovial γδ T Cells

**DOI:** 10.4049/jimmunol.1900451

**Published:** 2019-09-23

**Authors:** Cheryl Collins, Yuan Lui, Ana Mafalda Santos, Bryan A. Ballif, Anisha Mahalya Gogerly-Moragoda, Heather Brouwer, Robin Ross, Kuberan Balagurunathan, Sumana Sharma, Gavin J. Wright, Simon Davis, Ralph C. Budd

**Affiliations:** * Vermont Center for Immunology and Infectious Diseases, Department of Medicine, Larner College of Medicine, The University of Vermont, Burlington, VT 05405;; † Weatherall Institute of Molecular Medicine, John Radcliffe Hospital, Headington, Oxford OX3 9DS, United Kingdom;; ‡ Department of Biology, College of Arts and Sciences, The University of Vermont, Burlington, VT 05405;; § Department of Medicine, Brigham and Women’s Hospital, Boston, MA 02115;; ¶ Department of Medicinal Chemistry, University of Utah, Salt Lake City, UT 84112; and; ‖ Wellcome Sanger Institute, Cambridge CB10 1SA, United Kingdom

## Abstract

Lack of understanding of the nature and physiological regulation of γδ T cell ligands has considerably hampered full understanding of the function of these cells. We developed an unbiased approach to identify human γδ T cells ligands by the production of a soluble TCR-γδ (sTCR-γδ) tetramer from a synovial Vδ1 γδ T cell clone from a Lyme arthritis patient. The sTCR-γδ was used in flow cytometry to initially define the spectrum of ligand expression by both human tumor cell lines and certain human primary cells. Analysis of diverse tumor cell lines revealed high ligand expression on several of epithelial or fibroblast origin, whereas those of hematopoietic origin were largely devoid of ligand. This allowed a bioinformatics-based identification of candidate ligands using RNAseq data from each tumor line. We further observed that whereas fresh monocytes and T cells expressed low to negligible levels of TCR-γδ ligands, activation of these cells resulted in upregulation of surface ligand expression. Ligand upregulation on monocytes was partly dependent upon IL-1β. The sTCR-γδ tetramer was then used to bind candidate ligands from lysates of activated monocytes and analyzed by mass spectrometry. Surface TCR-γδ ligand was eliminated by treatment with trypsin or removal of glycosaminoglycans, and also suppressed by inhibition of endoplasmic reticulum–Golgi transport. Of particular interest was that inhibition of glycolysis also blocked TCR-γδ ligand expression. These findings demonstrate the spectrum of ligand(s) expression for human synovial Vδ1 γδ T cells as well as the physiology that regulates their expression.

## Key Points

- TCR-γδ tetramer identified ligand expression by flow cytometry.
- TCR-γδ ligands were induced on activated monocytes or T cells.
- Bioinformatics combined with mass spectrometry produced an overlapping list of 16 candidate ligands.

## Visual Abstract

**Figure:**
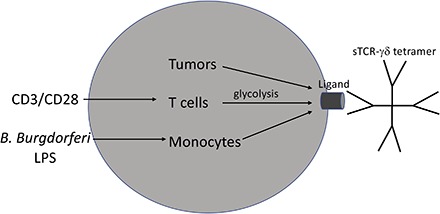

## Introduction

Full understanding of γδ T cell biology has been handicapped by ignorance of the ligands for most TCR-γδ. γδ T cells reside at mucosal and epithelial barriers and often accumulate at sites of inflammation with autoimmunity, infections, or tumors (1). Evidence suggests that γδ T cells provide protection against infections with bacteria, viruses, and protozoans and are generally beneficial in autoimmunity (1–17). In addition, a role for γδ T cells in the immune response against tumors in humans is evident from a seminal study reporting that intratumoral γδ T cells are the most favorable prognostic immune population across 39 cancer types in humans (18). γδ T cells are often highly lytic against transformed proliferative cells, infected cells, and infiltrating CD4^+^ T cells in inflammatory arthritis (9, 17, 19). They can produce a variety of cytokines including IFN-γ, TNF-α, and IL-17 (20), as well as insulin-like growth factor-1 (IGF1) and keratinocyte growth factor (KGF) that promote epithelial wound repair (21). These collective studies indicate that a principal function of γδ T cells is in response to tissue injury of various causes. It is, thus, not surprising that γδ T cells are often suggested to react to host components that are upregulated or exposed during proliferation or cell injury (22). As such, γδ T cells may function in tissue homeostasis and immunoregulation as much as in protection from infection. Yet in the vast majority of cases, little if anything is known regarding the nature of these self-components or whether they actually engage the TCR-γδ.

Whereas αβ T cells recognize proteins that are processed into peptides and presented on MHC molecules, the few proposed ligands for γδ T cells suggest that they recognize mostly intact proteins directly, without MHC restriction. This makes them highly attractive for immunotherapy. Despite the elaborate mechanisms that αβ T cells and B cells use to prevent autoreactivity, γδ T cells have been frequently reported to respond to autologous proteins. Furthermore, in contrast to other lymphocytes that maximize the potential diversity of their receptors, γδ T cells frequently show limitations in their diversity. Thus, human γδ T cells comprise a subset of Vδ2 T cells, the predominant γδ in peripheral blood that respond to prenyl phosphates and certain alkyl amines (23–25), and Vδ1 T cells, which do not respond to these compounds and often accumulate at epithelial barriers and sites of inflammation (1). A similar limited repertoire occurs in the mouse in which Vγ5Vδ1 cells colonize the epidermis, and a Vγ6Vδ1 subset colonizes the tongue, lung, and female reproductive tract (21, 26). This restricted repertoire implies that TCR-γδ ligands may also be limited. This may provide for a more rapid response and perhaps explain why, in contrast to αβ T cells and B cells, it is difficult to generate Ag-specific γδ T cells by immunization with a defined Ag.

Various ligands for γδ T cells have been proposed, although only a few have been confirmed to bind to TCR-γδ, and these lack any obvious similarity in structure. γδ T cells for which ligands have been identified include the murine γδ T cell clone G8, which recognizes the MHC class I–like molecules T10 and T22 (27), γδ T cells from mice infected with HSV that recognize herpes glycoprotein gl (28), a subset of murine and human γδ T cells that bind the algae protein PE (20), a human γδ T cell clone G115 that recognizes ATP synthase complexed with ApoA-1 (28), a human γδ T cell clone (Vγ4Vδ5) from a CMV-infected transplant patient that recognizes endothelial protein C receptor (EPCR) (29), and some human Vδ1 T cells that recognize CD1d-sulfatide Ags (30). However, to date no systematic process has been reported for determining the spectrum of human TCR-γδ ligands.

To provide an unbiased approach for the identification of candidate ligands for human γδ T cells, we produced a biotinylatable form of a soluble TCR-γδ (sTCR-γδ) from a synovial Vδ1 γδ T cell clone of a Lyme arthritis patient. The tetramerized sTCR-γδ was used in flow cytometry to identify various cell types that expressed candidate ligands. Initial analysis of 24 tumor cell lines identified a set of nine ligand-positive tumors, enriched for those of epithelial and fibroblast origin, and 15 ligand-negative tumors, largely of hematopoietic origin. In addition, ligand was not expressed by primary monocytes or T cells, although each could be induced to express ligand following their activation. Ligand expression was sensitive to trypsin digestion, revealing the protein nature of the ligands, and was also reduced by inhibition of glycolysis. These findings provide a framework and strategy for the identification of individual ligands for human synovial γδ T cells.

## Materials and Methods

### Production of a sTCR-γδ

**FIGURE 1.**
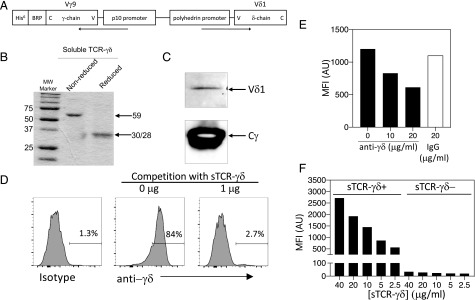
Production of human synovial sTCR-γδ. (**A**) pBACp10pH vector containing the δ-chain driven by the polyhedrin promoter and the γ-chain with hexa-His and biotinylation BRP sequences driven by the p10 promoter from γδ T cell clone Bb15 (Vγ9Vδ1). (**B**) Sample of nickel NTA column-purified sTCR-γδ analyzed by SDS-PAGE under reducing and nonreducing conditions, and stained with Coomassie Blue. (**C**) Immunoblot of sTCR-γδ stained with anti-Vδ1 or anti-Cγ. (**D**) γδ T cell clone Bb15 was stained with anti–TCR-γδ Ab in the absence or presence of competing sTCR-γδ. (**E**) The fibrosarcoma cell line 2fTGH was stained with the sTCR-γδ in the absence or presence of the indicated concentrations of anti-γδ Ab or control IgG. (**F**) Titration of sTCR-γδ staining of the positively staining tumor line 2fTGH or negatively staining line Daudi. Number inserts indicate percent positively staining cells. Findings are representative of three experiments.

Human synovial γδ T cell clones from a Lyme arthritis patient were produced as previously described (9, 31). One of these clones, Bb15, was chosen for production of the sTCR-γδ using modification of a previously reported procedure (32, 33). Both TCR chains were produced as a single transcript in a baculovirus vector. The pBACp10pH vector used contains two back-to-back promoters, p10 and polyhedrin (Fig. 1A). The p10 promoter is followed by multiple cloning sites for the γ-chain, and the polyhedrin promoter is followed by multiple cloning sites for the δ-chain. Downstream of the γ-chain, we placed a hexa-His tag for nickel column purification, followed by a biotinylation sequence for tetramerization. The γ-chain and δ-chain were PCR amplified using high fidelity polymerase (Deep Vent Polymerase; New England Biolabs). Both TCR chain sequences were verified following the initial PCR amplification as well as after insertion into the pBACp10pH vector. Virus encoding the sTCR-γδ was generated by cotransfection of Sf21 moth cells using the Sapphire baculovirus DNA and Transfection kit (Orbigen) with the sTCR pBACp10pH construct. Virus was harvested 6 d later and used as primary stocks (P1 stock). Two additional rounds of viral amplification, P2 and P3, were completed using midlog phase Sf21 cells (∼1.6 × 10^6^ cells/ml) allowed to adhere for 1 h before infecting at a multiplicity of infection of 0.01 or 0.1 with P1 and P2 stock, respectively. After 72 h of infection, culture medium was clarified by centrifugation (1000 × *g* for 10 min) and filtration (VacuCap 90PF 0.8/0.2 μm Supor membrane filter units; Pall, Westborough, MA) before storing in the dark at 4°C until use. Protein production occurred in 12-l batches of midlog phase (∼1.6 × 10^6^ cells/ml) Hi5 cells growing in suspension (0.5 l of culture in 1 l spinner flasks) and infected with P3 stock at a 1:50 dilution. Following 72 h of infection, cells were removed by centrifugation and filtration as described above. The filtered supernatant (∼12 l) containing secreted sTCR-γδ was concentrated to ∼100 ml before dialyzing against 1 l of nickel column loading buffer (20 mM NaPhosphate buffer, pH 7.4, 20 mM imidazole, 0.5 M NaCl) using a Pellicon diafiltration system with two 10K MWCO membranes (MilliporeSigma, Burlington, MA) back down to ∼100 ml. After system flushing, the final sample volume was ∼200 ml. It was then loaded onto loading buffer–equilibrated His-Trap HP columns (GE Healthcare, Little Chalfont, U.K.) at 100 ml per 2 × 5 ml columns. Columns were washed with at least 10 column volumes of loading buffer until baseline absorption was achieved. Bound proteins were eluted using a gradient from 20 to 500 mM imidiazole over 20 column volumes. Elution was monitored by absorbance at 280 nM, and 1 ml fractions were collected. Fractions containing the target protein were identified using SDS-PAGE gel analysis using Coomassie Blue. High purity (>95%) sTCR-γδ fractions were pooled, dialyzed against PBS (pH 7.4), and frozen at −80°C until used in future studies. Yields were typically ∼1.0–2.5 mg/l of culture. Purified sTCR-γδ was then biotinylated using a biotin-protein ligase system (Avidity) and tetramerized with streptavidin-PE (BioLegend) for FACS staining. Verification of TCR-γδ protein was confirmed by SDS-PAGE gel analysis using Coomassie Blue as well as immunoblot using Abs to Vδ1 or Cγ (Endogen).

### Flow cytometry

Cells were stained with either sTCR-γδ-PE (10 μg/ml) or negative controls that included streptavidin-PE (10 μg/ml), IgG-PE (10 μg/ml) (BioLegend), or a sTCRαβ-PE (a kind gift of Dr. M. Davis). Additional surface staining of T cells consisted of CD4, CD8, CD19, and CD25 (BioLegend). Live–Dead staining (BD Bioscience) was used to eliminate dead cells from analysis. Samples were run on an LSRII flow cytometer (Becton Dickinson).

### Purification and activation of human monocytes and T cells and cell lines

Human monocytes were purified from human PBMC using CD14-labeled magnetic beads, followed by column purification (Miltenyi Biotec) and then cultured in RPMI complete medium with 10% FCS in the absence or presence of either a *Borrelia burgdorferi* sonicate (10 μg/ml) or LPS (1 μg/ml; Sigma-Aldrich) for 18 h. To some cultures were added TNF-α (10 ng/ml) (BioLegend), anti-TNF-α (10 μg/ml) (BioLegend), IL-1β (10 pg/ml) (Invitrogen), or anti–IL-1β (5 μg/ml) (R&D Systems). Cells were then stained with the sTCR-γδ tetramer. T cells from PBMC were either used fresh or were activated with anti-CD3/anti-CD28 (each 10 μg/ml; BioLegend) + IL-2 (50 U/ml; Cetus) and propagated for 3 d. Cells were then stained with the sTCR-γδ tetramer. Human PBMC were obtained using an approved protocol from The University of Vermont Human Studies Committee. Verified cell lines were obtained from American Type Culture Collection. CHO cells deficient for glycosaminoglycans (GAGs) were derived as previously described (34).

### Bioinformatics analysis

Expression profiling (35) based on Illumina RNAseq technology (36) was used to characterize the transcriptomes of 22 of the 24 tumor cell lines examined (excluding bronchoepithelial cell line and 2fTGH). Expression data for all known genes (37) were generated, and those genes whose representation in tetramer-positive cell lines was significantly higher than in negative cell lines were considered as candidate ligands.

### Mass spectrometry analysis

Biotinylated sTCR-γδ was bound to avidin magnetic beads and then incubated with cell lysates from monocytes activated with *B. burgdorferi* sonicate. Magnetic beads alone, without TCR-γδ tetramer, with monocyte lysates served as a negative control. After 4 h, beads were washed five times, and bound proteins were then separated on polyacrylamide gels. Gel lanes for each sample type were cut into 12 identical regions and diced into 1-mm cubes. In-gel tryptic digestion was conducted on each region as previously described (38). Extracted peptides were subjected to liquid chromatography tandem mass spectrometry (38), except that the analysis was performed using an LTQ linear ion trap mass spectrometer (Thermo Fisher Scientific, Waltham, MA). Tandem mass spectra were searched against the forward and reverse concatenated human IPI database using SEQUEST, requiring fully tryptic peptides, allowing a mass tolerance of 2 Da and mass additions of 16 Da for the oxidation of methionine and 71 Da for the addition of acrylamide to cysteine. SEQUEST matches in the first position were then filtered by XCorr scores of 1.8, 2, and 2.7 for singly, doubly, and triply charged ions, respectively. Protein matches made with more than two unique peptides were further considered. This list had a peptide false discovery rate of <0.01%.

### Inhibition of glycolysis, transcription, translation, and endoplasmic reticulum–Golgi transport or trypsin or heparinases I–III treatment

Inhibition of glycolysis was performed using the 2-deoxyglucose (2-DG, 5 mM; Sigma-Aldrich) for 48 h. Transcription and translation were inhibited using, respectively, actinomycin D (5 μg/ml; ICN Biomedicals) or cycloheximide (1 μg/ml; MilliporeSigma) for 18 h. Endoplasmic reticulum (ER)–Golgi transport was blocked using brefeldin A (1:1000) or monensin (1:1400) (BD Bioscience) for 18 h. Cell surface protein digestion was performed using trypsin (Invitrogen) (1×; 5–10 min, 37°C.). GAGs were removed from cells by treatment with heparinases I–III (2 μU/ml) for 30 min in RPMI 1640 with no serum. The reaction was then stopped by the addition of PBS–BSA.

### Statistical analysis

The following statistical tests were used: unpaired Student *t* test when comparing two conditions, and one-way ANOVA with Sidak test for correction for multiple comparisons when comparing multiple variables across multiple conditions.

## Results

### Production of a human synovial sTCR-γδ

We previously produced a panel of synovial Vδ1 γδ T cells from Lyme arthritis patients (9, 31). A representative clone, Bb15 (Vδ1Vγ9), was selected from which to clone its TCR-γδ. The pBACp10pH vector has been used previously to produce murine sTCR-γδ tetramers (33). It contains two back-to-back promoters, p10 and polyhedrin, in which the p10 promoter is followed by multiple cloning sites for inserting the γ-chain, and the polyhedrin promoter is followed by multiple cloning sites for inserting the δ-chain (Fig. 1A). Downstream of the γ-chain we placed a hexa-His tag for purification, followed by a biotinylation BRP sequence for tetramerization with streptavidin-PE. Protein production was undertaken in Hi5 cells followed by purification using His-Trap HP columns. Fractions were analyzed by SDS-PAGE, and those with protein of the correct size were pooled, with yields typically of 1–2 mg/l of culture. A sample sTCR-γδ preparation is shown in Fig. 1B, stained with Coomassie Blue, showing bands of the expected size for the heterodimer under nonreducing (59 kDa) and reducing conditions (30/28 kDa for the γ- and δ-chains, respectively). The protein was stained by immunoblot with Abs to either Vδ1 or Cγ (Fig. 1C) and also blocked anti-γδ Ab staining of the synovial γδ T cell clones (Fig. 1D). The purified sTCR-γδ was then biotinylated and tetramerized with streptavidin-PE for use by flow cytometry. As an additional measure of specificity, sTCR-γδ tetramer staining of a fibrosarcoma tumor cell line (2fTGH) could be inhibited by anti-γδ Ab but not control IgG (Fig. 1E). Finally, staining of 2fTGH cells with the sTCR-γδ tetramer was dose dependent but did not increase with increasing dose on a negative tumor line, Daudi (Fig. 1F).

### Expression of sTCR-γδ candidate ligand(s) varies among cell lines

**FIGURE 2.**
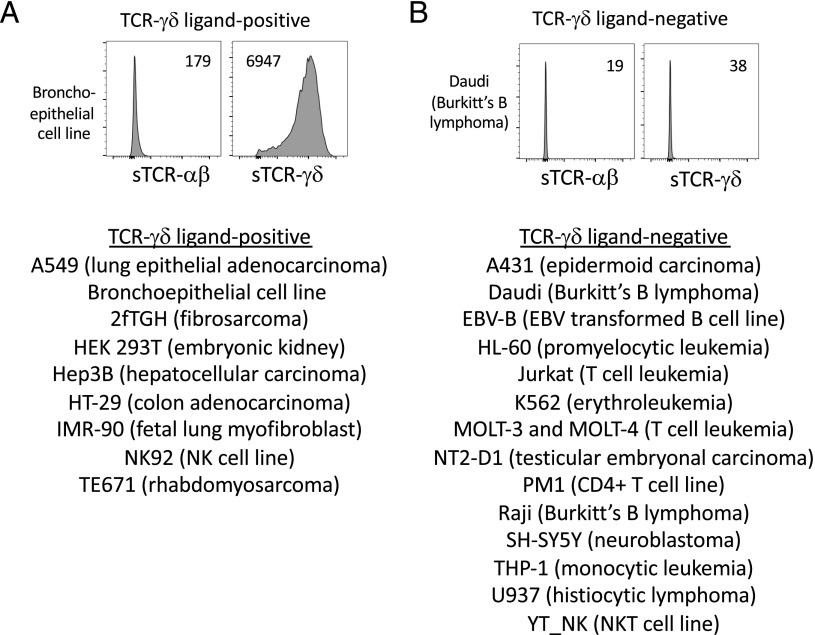
sTCR-γδ tetramer staining of a cell line panel. A panel of 24 diverse cell lines was stained with either sTCR-αβ or sTCR-γδ, gated on live cells, and examined by flow cytometry. Shown are examples of tumors representing either (**A**) positive staining or (**B**) negative staining with sTCR-γδ, with the complete list summarized below each example. Number inserts indicate mean fluorescence intensity of entire histogram. Findings are representative of four experiments.

We initially used the sTCR-γδ tetramer to screen a panel of 24 cell lines from a variety of cell types. None of the cell lines stained with the negative controls (IgG-PE, avidin-PE, or sTCR-αβ tetramer-PE), but the sTCR-γδ tetramer gave a spectrum of staining in which nine cell lines were strongly positive and the other cell lines manifested low to undetectable surface staining (Fig. 2). Of interest was that the positive group was enriched for cell lines of epithelial and fibroblast origin, cell types known to exist where γδ T cells are often found, such as skin, intestines, and synovium. With this information, expression profiling (35) using available RNAseq was used to characterize the transcriptomes of 22 of the 24 tumor cell lines (RNAseq on the bronchoepithelial and 2fTGH were not available). Expression data for all known genes (37) were generated, and those genes whose representation in tetramer-positive cell lines was significantly higher than in negative cell lines were considered to be candidate ligands. This produced an initial list of candidate ligands for sTCR-γδ (Supplemental Table I).

### Candidate sTCR-γδ ligands are sensitive to trypsin and reduced by inhibition of transcription, translation, ER–Golgi transport, or removal of GAGs

**FIGURE 3.**
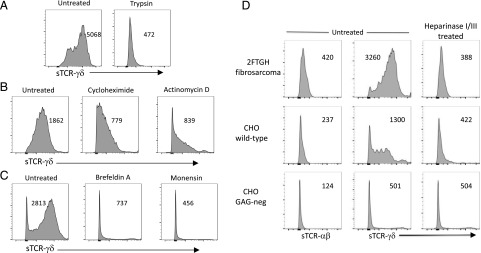
sTCR-γδ ligand is sensitive to protease, blockers of ER–Golgi transport, translation, or transcription and contains GAGs. The human bronchoepithelial cell line was either untreated or treated with (**A**) trypsin for 15 min, (**B**) untreated or treated for 18 h with cycloheximide or actinomycin D, or (**C**) untreated or treated for 18 h with brefeldin A or monensin. Cells were then stained with sTCR-γδ tetramer. (**D**) The 2fTGH fibrosarcoma cell line, wild-type CHO cells, or GAG-deficient CHO cells were either untreated or treated with a combination of heparinases I–III for 30 min and then stained with sTCR-γδ tetramer. Number inserts indicate mean fluorescence intensity of entire histogram. Findings are representative of three experiments.

We treated the positively staining cell lines with trypsin and noted a complete disappearance of surface staining, as exemplified for bronchoepithelial cells in Fig. 3A. Similar results were observed with two additional tumor lines. This supports the view that the TCR-γδ ligand contains a protein component essential for recognition by the receptor. We also observed no increase in sTCR-γδ tetramer staining of cells (C1R or HeLa) expressing CD1a, b, c, or d, nor with MICA/B (data not shown). Thus, at present there is no evidence that the synovial Vδ1 TCR-γδ ligand is one of these MHC class I–like molecules, at least bound to endogenous molecules from these particular cell lines.

We further determined that surface TCR-γδ ligand expression was reduced by inhibition of protein translation or transcription with, respectively, cycloheximide or actinomycin D (Fig. 3B). Surface ligand was also considerably reduced by inhibition of transport from the ER to Golgi using either brefeldin A or monensin (Fig. 3C). This further demonstrated the protein nature of candidate TCR-γδ ligands. Finally, we examined the extent to which GAGs contribute to ligand binding by TCR-γδ. This was tested in two ways. Initially, the ligand-positive fibrosarcoma cell line 2fTGH was either treated or not with heparinases I–III, which removes most GAGs. This considerably reduced sTCR-γδ tetramer staining (Fig. 3D). This was further supported by the observation that sTCR-γδ stained wild-type but not GAG-deficient CHO cells (Fig. 3D).

### sTCR-γδ ligands are expressed by activated monocytes

**FIGURE 4.**
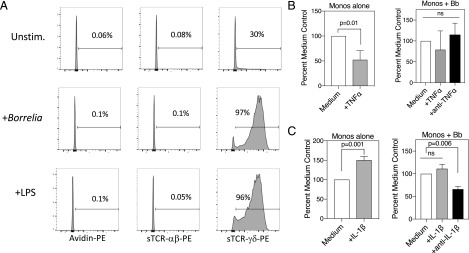
TCR-γδ ligand is induced on human monocytes following activation. (**A**) Freshly isolated monocytes were either unstimulated or activated with *B. burgdorferi* or LPS for 18 h and then stained with the indicated reagents and analyzed by flow cytometry. (**B** and **C**) Fresh monocytes or monocytes activated with *Borrelia* were incubated in the presence of medium alone or TNF-α or blocking anti-TNF-α (B) or IL-1β or blocking anti–IL-1β (C). Number inserts indicate percent positively staining cells. Error bars represent SEM. Findings are representative of four experiments.

In considering what primary cells might express ligand(s) for the sTCR-γδ, we first examined fresh monocytes, as we had observed previously that following their activation with *B. burgdorferi* or LPS, monocytes could activate the synovial γδ T cell clones (31). Consistent with these earlier findings, we observed that the sTCR-γδ tetramer did not stain freshly isolated human monocytes, but following 24 h activation with a sonicate of *B. burgdorferi* or LPS, there was a robust upregulation of sTCR-γδ tetramer staining (Fig. 4). The same cells did not stain with negative controls that included avidin-PE, IgG-PE, or a human sTCR-αβ tetramer-PE. Because activated monocytes are known to produce certain cytokines, particularly TNF-α and IL-1β, we examined the possible influence of these cytokines on ligand expression. Curiously, the low level of sTCR-γδ tetramer staining of fresh monocytes was reduced further with TNF-α, whereas ligand expression by *Borrelia*-activated monocytes was not affected by the further addition of TNF-α or blocking anti–TNF-α Ab (Fig. 4B). By contrast, IL-1β increased ligand expression by fresh but not activated monocytes, and blocking anti–IL-1β Ab partially inhibited ligand expression by activated monocytes (Fig. 4C). Thus, sTCR-γδ ligand expression appears to be partly regulated by certain monocyte-derived cytokines.

Given the induction of sTCR-γδ ligand expression by activated monocytes, we prepared lysates from *Borrelia*-activated monocytes and then used the biotinylated sTCR-γδ complexed with avidin magnetic beads as a bait. Following incubation with the monocyte lysates, the sTCR-γδ was isolated by magnetic purification and washed five times; bound proteins were separated on polyacrylamide gels, and gel slices were subjected to trypsin digestion and analyzed by mass spectrometry. Avidin magnetic beads alone incubated with monocyte lysates served as a negative control. This analysis yielded 291 unique proteins (Supplemental Table II). When compared with the list produced by the RNAseq bioinformatics approach of the tumor lines, 16 proteins were found in common (Supplemental Table III). Of interest is that two of these, Annexin A2 and heat shock protein 70, have previously been proposed as γδ ligands (39–41).

### sTCR-γδ ligands are expressed by activated T cells

**FIGURE 5.**
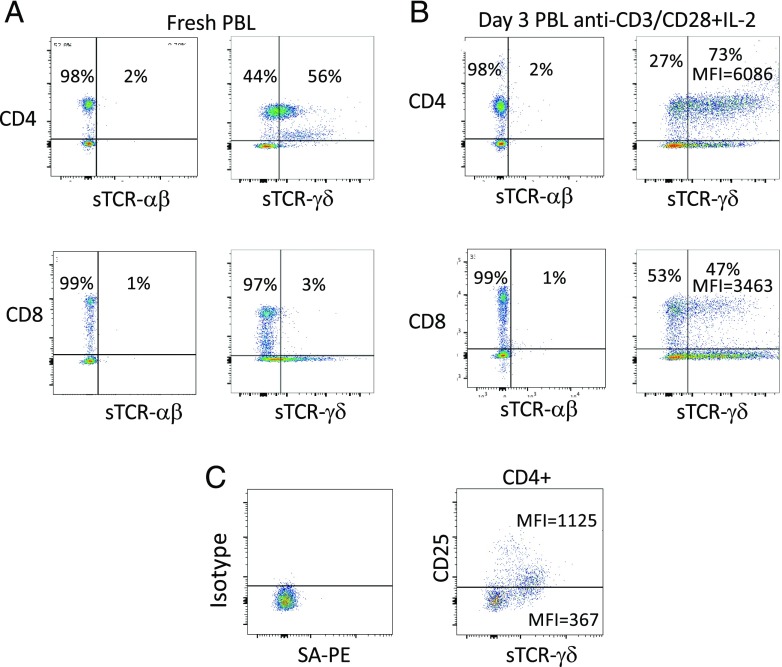
sTCR-γδ tetramer stains a subset of activated human T cells and Treg. PBL were stained with Abs to CD4 and CD8 as well as with sTCR-αβ tetramer-PE or sTCR-γδ tetramer-PE either (**A**) freshly isolated or (**B**) 3 d after activation with anti-CD3/CD28 + IL-2. Number inserts indicate the percentages of T cells staining negatively or positively with sTCR-γδ tetramer, as a portion of the total CD4^+^ or CD8^+^ subsets, as well as mean fluorescence intensity (MFI) in some cases. Findings are representative of six experiments. (**C**) Freshly isolated PBL were stained with anti-CD4, anti-CD25 or isotype control, and streptavidin-PE (SA-PE) or sTCR-γδ-PE. Shown are cells gated on CD4 expression. Number inserts indicate MFI of sTCR-γδ-PE staining for CD25^+^ and CD25^−^ subsets. Findings are representative of two experiments.

We further analyzed freshly isolated PBL from three individuals of various ages (28–66). This consistently revealed that fresh CD8^+^ T cells exhibited negligible sTCR-γδ staining, whereas a subset of fresh CD4^+^ T cells manifested modest levels of sTCR-γδ staining (Fig. 5A). In contrast to the freshly isolated T cells, following 3 d activation with anti-CD3/CD28 + IL-2, we observed that a subset of both CD4^+^ and CD8^+^ T cells now displayed high levels of sTCR-γδ staining (Fig. 5B). Both the proportion of cells expressing ligand and the density was higher on activated CD4^+^ T cells compared with CD8^+^ T cells. Given that in vitro–activated proliferating T cells express sTCR-γδ ligand, we considered that the subset of fresh CD4^+^ T cells expressing ligand might also represent a proliferative subset. One of the most rapidly proliferative T cell subsets in vivo is T regulatory cells (Treg) (42). Treg can be identified as a subset of fresh CD4^+^ T cells expressing CD25. Indeed, when we subset fresh human CD4^+^ T cells based on CD25 expression, sTCR-γδ tetramer staining was again observed preferentially by the CD25^+^ subset (Fig. 5C).

### TCR-γδ ligand expression is partly dependent upon glycolysis

**FIGURE 6.**
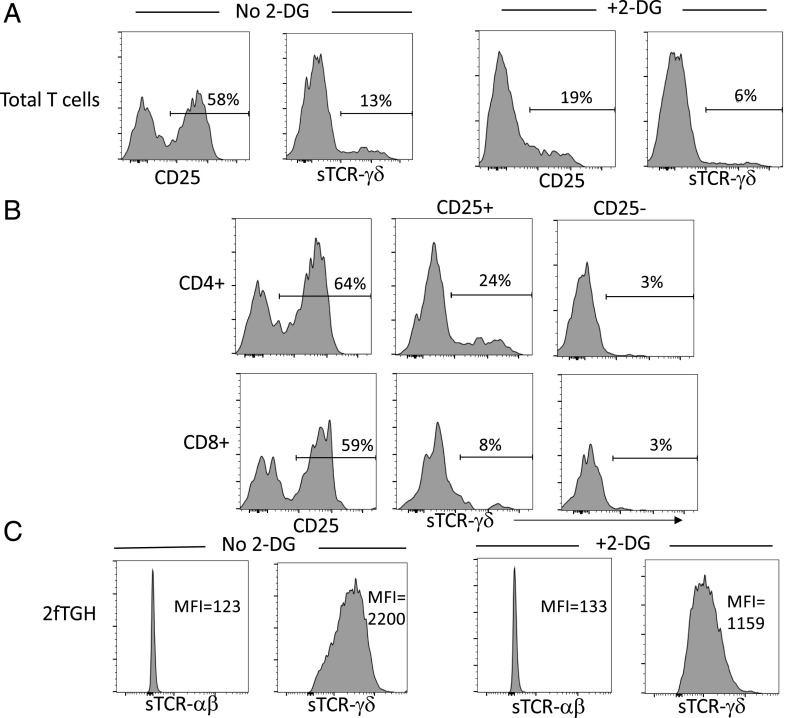
TCR-γδ ligand expression parallels glycolysis. (**A** and **B**) PBL were activated with anti-CD3/CD28 + IL-2 in the absence or presence of 2-DG (5 mM). On day 3, cells were stained with Abs to CD4, CD8, CD25, and sTCR-γδ tetramer-PE. Shown in (A) are the levels of CD25 and TCR-γδ ligand without or with 2-DG. Shown in (B) is the expression of TCR-γδ ligand in CD4^+^ or CD8^+^ subsets based on surface CD25. (**C**) 2fTGH cells were cultured for 48 h in either regular medium or medium plus 2-DG (5 mM). Cells were then stained with TCR-αβ or TCR-γδ. Number inserts indicate mean fluorescence intensity (MFI) of sTCR-γδ-PE staining. Findings are representative of three experiments.

The finding that fresh monocytes and T lymphocytes expressed low to negligible levels of sTCR-γδ ligand(s), but upregulated expression following activation, raised the possibility that this might reflect the known induction of glycolysis following activation of T cells, monocytes, or dendritic cells (43, 44) and the resultant synthetic capacity promoted by glycolysis (45). This notion is supported by the fact that ligand-expressing Treg are also highly glycolytic (42). We thus examined this question in two ways. First, we exposed activated T cells to 2-DG, an inhibitor of glycolysis. This reduced expression of both CD25 and sTCR-γδ ligand (Fig. 6A). Second, we distinguished between activated T cells on day 3 based on their expression of CD25, as this identifies cells responsive to IL-2 and are hence most glycolytic (45). As shown in Fig. 6B, CD25^+^ T cells expressed sTCR-γδ ligand whereas the CD25^−^ subset was devoid of ligand expression. Of further note is that within the CD25^+^ subset, CD4^+^ T cells again expressed more ligand than CD8^+^ T cells (Fig. 6B). We extended this analysis to the ligand-positive tumor 2fTGH and observed that 2-DG also resulted in reduced ligand expression in these cells (Fig. 6C).

## Discussion

To our knowledge, the current findings provide the first unbiased characterization of the spectrum of ligand expression for human synovial Vδ1 γδ T cells. The range of ligand expression may reflect the various locations and seemingly diverse functions attributed to γδ T cells. For example, ligand induction by *B. burgdorferi*– or LPS-activated monocytes parallels their known ability to activate synovial γδ T cell clones (9, 31). In addition, ligand expression by fresh CD4^+^ but not CD8^+^ T cells also correlates with our previous observations that Lyme arthritis synovial γδ T cells suppress by cytolysis the expansion of synovial CD4^+^ but not CD8^+^ T cells in response to *B. burgdorferi* (9). Finally, defining the spectrum of tumor cell types that express TCR-Vδ1 ligands may help explain which tumors contain Vδ1 γδ T cells and impact their effectiveness as immunotherapy. The collective findings are also most consistent with the view that γδ T cells respond to self-proteins as much as or possibly more than foreign proteins. Although these results were obtained using a sTCR-γδ tetramer from a single synovial γδ T cell clone, the fact that it shares a common Vδ1 chain found on most synovial γδ T cells (9), as well as γδ T cells found in intestinal epithelium (1, 10, 21), several tumors (18), and cells expanded in PBL following certain infections such as HIV (46, 47) and CMV (29), suggests the possibility that Vδ1 γδ T cells from these other sources may share a common physiology of ligand expression.

Previous studies of ligands for murine and human γδ T cells have come largely from the identification of individual molecules that activate a specific γδ T cell clone (27–30). Although this has been successful in some instances, the current study applied a broader approach of using a sTCR-γδ tetramer in an unbiased fashion to identify the spectrum of ligand expression and how they are regulated. This approach also provided two independent methods by which to identify candidate ligands. One method used RNAseq transcriptome analysis from 22 tumor cell lines to match genes increased in positively staining tumors and decreased in negatively staining tumors. The second approach used the sTCR-γδ tetramer as a bait to bind ligands from lysates of activated monocytes and then identify the bound proteins by mass spectrometry. It is of considerable intertest that among these two sets of candidate ligands were 16 in common, two of which, Annexin A2 and heat shock protein 70, have been previously proposed as ligands for γδ T cells (39–41). By contrast, surface sTCR-γδ tetramer binding was eliminated by treatment with trypsin or removal of GAGs, and also suppressed by inhibition of ER–Golgi transport, suggesting the involvement of a combination of protein and GAGs in tetramer binding. Future studies will explore through knockdown and transfection methods whether any of the candidate ligands we have identified activate the original γδ T cell clone and the extent to which GAG/glycoprotein binding may or may not be a confounder.

Although the findings thus far have not determined whether there is one or several synovial Vδ1 TCR-γδ ligands, they do provide a framework for understanding the distribution and regulation of ligand expression, which is critical for better understanding of γδ T cell biology. For example, γδ T cells have been implicated in the defense against a variety of infections (2–7), which is consistent with our finding that different TLR agonists induce TCR-γδ ligand expression on monocytes. Similar studies using a murine sTCR-γδ also found ligands induced with bacterial infection (21). In addition, γδ T cells have been found to generally alleviate various autoimmune models (12–15), which may be consistent with the expression of ligand by a subset of activated CD4^+^ T cells.

The induction of TCR-γδ ligand expression by activation of primary monocytes or T cells, as well as ligand expression by a variety of highly proliferative tumor cell lines, suggested that the metabolic state of cells may influence their ability to express TCR-γδ ligands. Activation of monocytes and T cells is known to induce a metabolic switch to glycolysis to provide the synthetic capacity for proliferation (43, 44). In addition, Treg, which are known to be glycolytic in vivo (42), spontaneously expressed ligand. Moreover, most tumors are highly glycolytic, and the inhibition of glycolysis in these cells also reduced ligand expression. Collectively, these findings suggest that some γδ T cells may function to survey and regulate highly proliferative cells.

It is of some interest that the cell lines bearing high levels of TCR-γδ ligand expression were enriched for those of epithelial and fibroblast origin, because Vδ1 γδ T cells are typically found at epithelial barriers, such as skin or intestinal epithelium, as well as in inflamed synovium, which is rich in fibroblasts (48). By contrast, sTCR-γδ ligand expression was noticeably absent from most cell lines of hematopoietic origin. The spectrum of cell line staining with the human synovial sTCR-γδ also bears considerable similarity to previous results using a murine sTCR-γδ, which strongly stained epithelial and fibroblast tumors, and less well tumors of hematopoietic origin (33). These same murine sTCR-γδ also stained macrophages activated by TLR2 or TLR4 stimuli, similar to our findings with monocytes activated by *Borrelia* or LPS (49). Furthermore, staining of macrophages by the murine sTCR-γδ was also not affected by the absence of β2-microgloublin, suggesting little or no contribution of ligand by classical or nonclassical MHC class I molecules. This agrees with our findings that the human synovial sTCR-γδ tetramer staining was not affected by the presence or absence of CD1 or MICA/B molecules.

The findings in this study were made using primary cells and tumor cell lines. Future studies will attempt to extend these results to analyses of sTCR-γδ tetramer histologic staining of primary tissues as well as tumors and inflamed synovium to determine the spectrum of TCR-γδ ligand expression at these sites. Screening primary tumors for binding of sTCR-γδ tetramer may also help identify tumors that may benefit from immunotherapy with Vδ1 γδ T cells. In addition, identifying the ligands in inflamed synovium or intestinal epithelium will provide therapeutic strategies for manipulating the function of infiltrating γδ T cells.

## Supplementary Material

Data Supplement
