# Supplementary material for: Detection of Cell Surface Ligands for Human Synovial γδ T Cells
Source: J Immunol. 2019 Sep 23;203(9):2369–76. doi: 10.4049/jimmunol.1900451 (PMC6804759; doi:10.4049/jimmunol.1900451)
Supplement: Data Supplement [file JI_1900451.zip › JI_1900451_Supplemental_Table_3.pdf]

**Supplemental Table 3. sTCR-γδ candidate ligands common to both RNAseq Bioinformatics and Mass Spectrometry**

| **Ensembl Gene ID** | **EntrezGene ID** | **HGNC symbol** | **Protein** |
|---|---|---|---|
| ENSG00000134575 | 53 | ACP2 | acid phosphatase 2 |
| ENSG00000182718 | 302 | ANXA2 | annexin A2 |
| ENSG00000168374 | 378 | ARF4 | ADP ribosylation factor 4 |
| ENSG00000198668 | 805 | CALM1 | calmodulin 1 |
| ENSG00000160014 | 805 | CALM3 | calmodulin 3 |
| ENSG00000127022 | 821 | CANX | calnexin |
| ENSG00000134371 | 79577 | CDC73 | cell division cycle 73 |
| ENSG00000148180 | 2934 | GSN | gelsolin |
| ENSG00000166598 | 7184 | HSP90B1 | heat shock protein 90 beta family member |
| ENSG00000204388 | 3303 | HSPA1B | heat shock protein family A (HSP 70) member 1B |
| ENSG00000133816 | 9645 | MICAL2 | microtubule associated monooxygenase |
| ENSG00000166794 | 5479 | PPIB | peptidylprolyl isomerase B |
| ENSG00000135048 | 23670 | TMEM2 | transmembrane protein 2 |
| ENSG00000140416 | 7168 | TPM1 | tropomyosin 1 |
| ENSG00000035403 | 7414 | VCL | vinculin |
| ENSG00000164924 | 7534 | YWHAZ | tyrosine 3-monooxygenase |
